# Surgery's Debt to the Late Sir Henry Butlin

**Published:** 1912-02-03

**Authors:** 


### SURGERY'S DEBT TO THE LATE SIR HENRY BUTLIN.

The death of Sir Henry Trentham Butlin, Bart., creates a gap in the medical profession in this country which will not, we think, be easily or immediately filled. The deceased surgeon combined in himself several of those virtues which we are accustomed, rightly or wrongly, to regard as typically British; together with others which are as well exemplified abroad as at home. He had therefore, even before he was voted to the high honour of the Presidency of the Royal College of Surgeons, a position in the esteem of practitioners and in the affections of his patients which few are privileged to gain. In consultation with Butlin, medical men found themselves working with one whose intellect was never allowed to overrule his heart or his conscience. The acknowledged highest authority in Britain on those departments of surgery to which he especially devoted himself, he would never sanction or suggest any treatment unless he felt convinced that it was in the interests of the patient. No operation would he perform, even when urged to do so by patient or doctor or friends, if he thought the prospect of ultimate success was smaller than the risk involved. There are, fortunately, many men as high-minded among British surgeons, but where Butlin stood pre-eminent was in creating among total strangers instant confidence in his absolute honesty. When he advised operation, he made it perceptible to everyone that he did so from perfectly unselfish motives. Never was his judgment allowed to be swayed by any possible prospect of profit for himself; never by any craving for the advertisement which some operators contrive to get by attempting the injudicious or the impossible. These qualities are, of course, not unique; but in Butlin they were combined with powers of observation and diagnosis, with an originality of mind and outlook, with a technical skill in operating, and with a capacity for hard work, which were of themselves bound to place him in the front rank of operating surgeons. Receptive of new ideas he was always; yet he could discriminate the useless fad from the genuine discovery, and reject the former while adopting the latter. His own researches brought him recognition from near and far; and on the tongue and larynx, especially malignant disease of those regions, his opinion was regarded as the best that could be got. Space prevents any full account here of his published researches; but the ripe fruits of his genius and experience are well seen in his quite recent papers on *Unicellula Cancri* (see The Hospital, December 16, 1911, p. 277), and in those on cancer of the tongue, published in the *British Medical Journal* in January and February, 1909.

In the latter year he became President of the Royal College of Surgeons; and a year later he was simultaneously Chairman of the British Medical Association. In both capacities he worked hard and without a thought of himself, though even then there were warning symptoms of the pulmonary tuberculosis to which he succumbed. In the autumn of last year he resigned the Presidency of the College of Surgeons after two years of service as able and distinguished as that of the most famous of his predecessors; but the relief thus gained was insufficient to enable him to overcome the disease. The details of Butlin's career at St. Bartholomew's and the other hospitals with which he was connected are dealt with elsewhere; it is as a man of winning personality, unblemished honour, and scientific eminence combined with excellent commonsense, that one thinks of him now that he has left us.

